# 99mTc-HMPAO-labeled leukocyte SPECT/CT and transthoracic echocardiography diagnostic value in infective endocarditis

**DOI:** 10.1007/s10554-018-1487-x

**Published:** 2018-10-31

**Authors:** Katarzyna Holcman, Wojciech Szot, Paweł Rubiś, Agata Leśniak-Sobelga, Marta Hlawaty, Sylwia Wiśniowska-Śmiałek, Barbara Małecka, Andrzej Ząbek, Krzysztof Boczar, Agnieszka Stępień, Piotr Podolec, Magdalena Kostkiewicz

**Affiliations:** 1 Department of Cardiac and Vascular Diseases, Jagiellonian University Medical College, John Paul II Hospital, Pradnicka 80, 31-202 Kraków, Poland; 2 Department of Nuclear Medicine, John Paul II Hospital in Krakow, Kraków, Poland; 3 Department of Hygiene and Dietetics, Jagiellonian University Medical College, Kraków, Poland; 4 Department of Electrocardiology, Jagiellonian University Medical College, John Paul II Hospital, Kraków, Poland

**Keywords:** Infective endocarditis, Radiolabeled leukocytes, 99mTc-HMPAO, SPECT/CT, Echocardiography

## Abstract

Infective endocarditis (IE) is a life-threatening disease, establishing a diagnosis is often challenging. The aim of this prospective study was to evaluate and compare the diagnostic performance of the combined use of single photon emission tomography and computed tomography with technetium99m-hexamethylpropyleneamineoxime—labeled leukocytes (99mTc-HMPAO-SPECT/CT) with transthoracic echocardiography (TTE) in patients with suspected IE. We enrolled 40 consecutive patients (12 females, 28 males, mean age: 58.6 ± 18) with suspected IE in the years 2015–2016. All patients underwent clinical evaluation, TTE and 99mTc-HMPAO-SPECT/CT for the assessment of lesions typical for IE. Scans were evaluated for the presence and location of increased radioactivity foci, corresponding to the accumulation of radiolabeled leukocytes in inflammatory lesions. After 6 months, the patients were re-evaluated clinically and with TTE. Final IE diagnosis was established in 14 (35%) patients. Lesions typical for IE were shown in 28 (70%) TTEs and 16 (40%) 99mTc-HMPAO-SPECT/CTs. The latter tests were characterized by 90% accuracy, 93% sensitivity, 88% specificity, 96% negative predictive value (NPV), 81% positive predictive value (PPV). TTE demonstrated 60% accuracy, 93% sensitivity, 42% specificity, 92% NPV, and 46% PPV. 99mTc-HMPAO-SPECT/CT was characterized by a lower number of false-positive results compared to TTE (3 vs. 15). In patients with suspected IE, 99mTc-HMPAO-SPECT/CT yields a smaller number of false-positive results, significantly higher diagnostic accuracy, specificity and PPV than TTE. It helps to differentiate IE infectious and sterile echocardiographic lesions and reduces by 27% the number of misdiagnosed IE classified in the ‘possible IE’ category by modified Duke Criteria.

## Introduction

Infective endocarditis (IE) is a life-threatening disease with heterogeneous clinical manifestations. It has become one of the four most common life-threatening infection syndromes [1]. Despite introducing therapeutic advances in the last 20 years, the mortality rates associated with IE have not decreased [2]. Its heterogeneous nature, stemming from the diverse number of causative pathogens involved, as well as underlying cardiac comorbidities, hamper any straightforward diagnosis. IE consists of a variety of different types depending on the presence of intra-cardiac foreign materials, such as native valve IE (NVE), prosthetic valve IE (PVE) and device-related IE (CDRIE) [2]. The probability of IE is assessed with the modified Duke Criteria. Microbiology and echocardiography (transthoracic-TTE, transoesophageal-TEE) play key roles in the initial diagnosis and management. Currently, there are no reliable, distinct biomarkers of IE [3]. TTE has a sensitivity for NVE and PVE diagnosis of 70% and 50%, respectively [4, 5]. Even though specificity for the use of TTE has been reported to reach 90%, echocardiogram without abnormalities typical for IE does not exclude infections and there are many factors hindering its interpretation [6]. Echocardiographic evaluation is particularly difficult in the case of pre-existing valvular lesions, valvular prostheses or intracardiac devices. On the other hand, false-positive results are associated with thrombi, Lambl’s excrescences, cusp prolapse, chordal rupture, fibroelastoma, and Libman–Sacks lesions [6].

Single photon emission tomography with technetium99m hexamethylpropyleneamine oxime-labelled autologous leukocytes (99mTc-HMPAO-SPECT/CT) is an emerging technique in IE diagnostics. The accumulation of time-dependent radiolabelled leucocytes (99mTc-HMPAO-WBC) is registered to evaluate in vivo inflammatory lesions [7]. Recent European Society of Cardiology (ESC) guidelines have introduced 99mTc-HMPAO-SPECT/CT to IE diagnostics in selected clinical situations [6]. The main added value of this technique is its high specificity and detection of peripheral embolic events. Given recent published data, nuclear imaging was introduced into the PVE diagnostic pathway. 99mTc-HMPAO-SPECT/CT should be used when the diagnosis of PVE remains only ‘possible’ or even ‘rejected’ but with a persisting high level of clinical suspicion, either for the diagnosis of cardiac involvement or for imaging embolic events. In addition, 99mTc-HMPAO-SPECT/CT has been shown to have a potential role in CDRIE diagnostics; however, the data produced via this technique has not been sufficient to warrant its inclusion in the diagnostic pathway. Currently, 99mTc-HMPAO-SPECT/CT may be considered an additive tool in patients with suspected CDRIE, positive blood cultures and non-diagnostic echocardiography. This technique was reported to be useful for detecting left-ventricular-assist device and prosthetic vascular graft infections [8, 9]. Still, 99mTc-HMPAO-SPECT/CT has not yet found its place in American Heart Association and American College of Cardiology guidelines [1, 10].

Insufficient data on the properties of 99mTc-HMPAO-SPECT/CT compared to TTE has driven the need to address this issue. The aim of this prospective study was to evaluate and compare the results and diagnostic utility of 99mTc-HMPAO-SPECT/CT and TTE in patients with suspected IE.

## Materials and methods

### Study population

Over the period 2015–2016, we enrolled 40 consecutive adults with suspected IE based on the standard medical diagnostic process. Exclusion criteria included: pregnancy, lactation, renal replacement therapy, neutropenia (below 1500 cells per microliter), and diagnosed coexisting neoplastic disease. All patients underwent clinical evaluation with modified Duke Criteria assessment. Workup included white blood cell (WBC) count, C-reactive protein (CRP), procalcitonin (PCT) and three sets of peripheral venous blood samples for microbiological diagnostics. According to the 99mTc-HMPAO-SPECT/CT patients were stratified into those with the presence of intracardiac foci (group 1, n = 16) and those without (group 2, n = 24).

### Echocardiography

Participants had TTE for the evaluation of lesions typical for IE, namely, vegetation, abscess, new dehiscence of a prosthetic valve, pseudoaneurysm, perforation, fistula, valve aneurysm. Exams were performed with Philips EPIQ7 (the Netherlands) device according to ESC guidelines at the time of patient enrolment, and then after a 6-month follow up period [4, 6].

### Scintigraphy

**Fig. 1:**
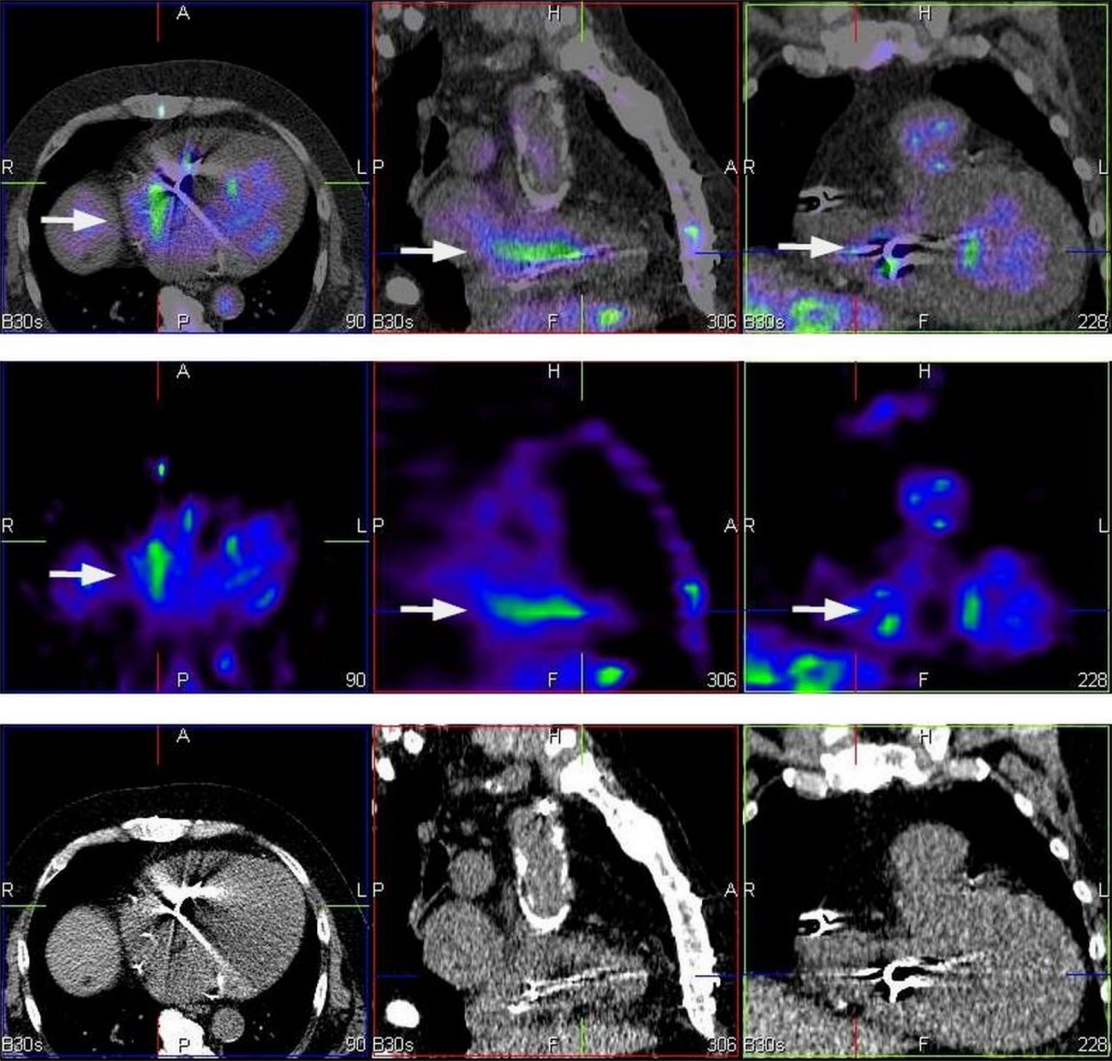
99mTc-HMPAO-SPECT/CT—study in three projections axial (left), sagittal (middle) and coronal (right). Bottom panel presents CT scans. Corresponding SPECT sections are presented in middle row and fusion images (99mTc-HMPAO-SPECT/CT) are placed in upper row. 99mTc-HMPAO-SPECT/CT shows an accumulation of radiolabeled leucocytes in the vicinity of an implanted electrode (arrows). In this case tracer uptake is consistent with CDRIE

Radiolabelling procedures were carried out in line with the European Society of Nuclear Medicine guidelines (dose: 370–740 MBq) [7]. Scans were performed in supine position after 4–6 h and 20–24 h following intravenous 99mTc-HMPAO-WBC injection [11]. Images were acquired using a dual-head, variable-angle Siemens Symbia T16 SPECT/CT gamma camera (Germany). Computed tomography (CT) attenuation-corrected and noncorrected SPECT images were assessed in the coronal, transaxial, and sagittal planes, as well as in tridimensional maximal-intensity projection cine mode. Matching pairs of CT transmission and radionuclide emission images were fused for hybrid imaging (Fig. 1).

The images obtained were evaluated for the presence and location of foci of increased radioactivity, which correspond to the accumulation of radiolabelled leukocytes in inflammatory lesions. Scans were classified as negative when no sites of pathological uptake were observed, or as positive in the cases where there was at least one intracardiac focus of abnormal uptake, characterized by typical time-dependent radioactivity pattern [12]. The extent of infection was assessed with regards to the involvement of the native valves, heart valve prostheses, endocardium, cardiac device lodge and electrodes [12–14]. The exam included extracardiac inflammatory foci evaluation. Exams were evaluated by two blinded, experienced nuclear medicine specialists.

### Follow-up

All patients were followed for 6 months. After that period an outpatient visit was scheduled for clinical re-evaluation and TTE.

### Statistical analysis

The Shapiro–Wilk test was used to assess conformity with a normal distribution. The continuous variables were compared between groups using Student’s t-test for mean values, and the Mann–Whitney U test for distribution. Categorical variables were analysed using the χ^2^ test or Fisher’s exact test as appropriate. The results of 99mTc-HMPAO-SPECT/CT, TTE, modified Duke Criteria were compared with the final clinical IE diagnosis, taking into account microbiological testing. In all cases, there was a follow-up after 6 months and subsequent outpatient visit, with clinical re-evaluation and TTE being performed for IE final diagnosis verification. The sensitivity, specificity, accuracy, negative predictive value (NPV), and positive predictive value (PPV) were calculated based on the final diagnosis and follow-up observation, with 95% confidence intervals (CIs), and compared using the kappa statistic for inter-rater reliability evaluation. Statistical analyses were performed using SPSS 23.0 (SPSS Inc., Chicago, IL, USA). p-values < 0.05 were accepted as statistically significant.

## Results

### Study population

The characteristics of patients (12 females, 28 males) are shown in Table 1. There were no differences between groups regarding the demographic and clinical profile, CRP and PCT values (p > 0.05). Most study participants (80%) had a cardiac implantable electronic device. Overall 15% of patients presented with a pocket site infection. Implanted prosthetic heart valves were present in 22.5% of the study population. According to the modified Duke Criteria, 7 patients (17.5%) had definite IE, 26 (65%) had possible IE, and 7 (17.5%) did not have IE.

**Table 1:** Characteristics of patients included in the study

Variable	Value^a^	Group 1^a^ (n = 16)	Group 2^a^ (n = 24)	p value
Age (years)	58.6 ± 18	59 ± 18	58 ± 19	0.98
Gender				
Female	12 (30%)	4 (25%)	8 (33%)	0.57
Male	28 (70%)	12 (75%)	16 (67%)	
Body mass (kg)	76 ± 15.7	71.7 ± 15.9	80.2 ± 14.7	0.15
Body high (cm)	171 ± 8.2	170 ± 7.5	172 ± 9	0.71
Body mass index (kg/m^2^)	25.9 ± 4.3	24.5 ± 4.2	27.4 ± 4.1	0.08
Body surface area (m^2^)	1.88 ± 0.22	1.82 ± 0.22	1.93 ± 0.21	0.20
Heart failure (NYHA I/II/III/IV)	30 (75%) (5/10/11/4)	14 (87%) (2/5/6/1)	16 (67%) (3/5/5/3)	0.46
Diabetes mellitus	9 (22.5%)	4 (25%)	5 (21%)	0.76
Arterial hypertension	20 (50%)	5 (31%)	15 (63%)	0.053
Atrial fibrillation	12 (30%)	6 (38%)	6 (25%)	0.40
Chronic liver disease	7 (17.5%)	3 (19%)	4 (17%)	0.79
Cardiovascular implantable electronic device present	32 (80%)	15 (94%)	17 (71%)	0.08
Pacemaker	18 (45%)	7 (44%)	11 (46%)	
ICD	9 (22.5%)	6 (38%)	3 (12%)	
CRT	5 (12.5%)	2 (12%)	3 (12%)	
Years since implantation of device	8 ± 6.9	7.6 ± 6.2	8.1 ± 7.4	0.93
Implanted prosthetic heart valve	9 (22.5%)	4 (25%)	5 (21%)	0.76
Aortic mechanical valve	4 (10%)	1 (6%)	3 (12%)	
Biological aortic valve	3 (7.5%)	1 (6%)	2 (8%)	
Mitral mechanical valve	2 (5%)	2 (12%)	0	
Mitral biological valve	1 (2.5%)	1 (6%)	0	
Years since implantation of valve	10 ± 7.4	10.8 ± 10.5	7.5 ± 6.2	0.58
Maximum body temperature (°C)	37.59 ± 1	37.5 ± 0.82	37.6 ± 1.2	0.87
Fever	17 (42.5%)	7 (44%)	10 (42%)	0.89
Number of leukocytes in peripheral blood (10^9^/l)	9.83 ± 4.31	10.51 ± 4.69	9.37 ± 4.08	0.22
Number of neutrophils in peripheral blood (10^9^/l)	7.14 ± 4.12	7.63 ± 4.9	6.81 ± 3.58	0.58
C-reactive protein (mg/l) [median, q1–q3]	52.76 ± 0.29 [15.5 (3.0–58.5)]	65.48 ± 95.7 [30 (2.5–67)]	44.28 ± 69.05 [13 (3–49)]	0.57
Procalcitonin (ng/ml) [median, q1–q3]	2.45 ± 7.93 [0.05 (0.05–0.17)]	2.89 ± 10.96 [0.05 (0.05–0.25)]	2.16 ± 5.3 [0.05 (0.05–0.21)]	0.24
Left ventricle ejection fraction (%)	44.6 ± 17.8	42 ± 18	47 ± 18	0.54
Left ventricle end-diastolic diameter (mm)	56.26 ± 11.9	57 ± 14	56 ± 11	0.98
Right ventricle proximal outflow tract diameter (mm)	33.2 ± 7	34 ± 7	32 ± 7	0.49
Tricuspid annular plane systolic excursion (mm)	18.1 ± 5.6	17 ± 6	19 ± 5	0.71
Right atrium area (cm^2^)	22.2 ± 9.4	25 ± 12	20 ± 6	0.36
Left atrium area (cm^2^)	24.8 ± 7.7	25 ± 9	25 ± 6	0.82
Pericardial effusion	4 (10%)	2 (12%)	2 (8%)	1.00
Echocardiographic lesions typical for IE	28 (70%)	14 (87%)	14 (58%)	0.04
Echocardiography positive for IE—mitral valve	3 (7.5%)	1 (6%)	2 (8%)	1.00
Echocardiography positive for IE—aortic valve	6 (15%)	2 (12%)	4 (16%)	0.72
Echocardiography positive for IE—tricuspid valve	3 (7.5%)	1 (6%)	2 (8%)	1.00
Echocardiography positive for IE—pulmonic valve	1 (2.5%)	1 (6%)	0 (0%)	1.00
Vegetations	24 (60%)	12 (75%)	12 (50%)	0.12
Vegetations within an electrode	20 (50%)	12 (75%)	8 (30%)	0.01
Valvular vegetations	10 (24%)	4 (25%)	6 (25%)	1.00
New severe valve regurgitation (valve perforation or prosthetic valve dehiscence)	3 (7.5%)	1 (6%)	2 (8%)	1.00
Perivalvular abscess	1 (2.5%)	0 (0%)	1 (4%)	1.00
Valve aneurysm	1 (2.5%)	1 (6%)	0 (0%)	1.00
Extracardiac infectious foci in 99mTc-HMPAO-SPECT/CT	19 (47.5%)	7 (44%)	12 (50%)	0.69

According to the 99mTc-HMPAO-SPECT/CT patients were stratified into those with the presence of intracardiac foci (group 1) and those without (group 2)

^a^Data are given as a number (percentage) for categorical data and as mean value ± one standard deviation or median (IQR) for continuous variable

Overall, 60% of blood cultures were negative. In the remaining patients with positive blood cultures, Staphylococci were the most common causative pathogens (10 patients, 63%), including 25% *Staphylococcus aureus* infections. In other cases, infection was caused by: Enterococci (19%), Klebsiella (19%), Streptococci (6%), Escherichia (6%), Proteus (6%), and Candida (6%). Overall, 45% of patients developed systemic inflammatory response syndrome (SIRS), 15% developed sepsis and 6% developed septic shock requiring inotropic support.

### Transthoracic echocardiography

Lesions typical for IE in TTE were present in 70% of patients. Most often there was vegetation (86%), new valvular regurgitation (14%), and annular abscess (4%). Any echocardiographic features diagnostic for IE associated with heart valves were observed in 35% of participants. Based on the TTE assessment, there was a suspicion of PVE in seven cases (18%). Vegetation within the intracardiac portion of electrodes was observed in 62.5% of patients with implantable cardiac devices.

### Scintigraphy

40% of 99mTc-HMPAO-SPECT/CTs classified as positive for IE and 5% showed isolated local device infection (LDI). The most common type of radiolabelled leukocyte accumulation consistent with IE was CDRIE, which accounted for 68.8% of the positive scans. Scintigraphy exams showed increased tracer uptake within native valves in 3 (7.5%) patients and within prosthetic valves IE was detected in 2 (5%) patients. Among the native valves and valve prostheses, aortic valve involvement was usually observed. Involvement of the tricuspid valve and pulmonic valve was less frequently encountered. Extracardiac foci of increased radioactivity were observed in 47.5% of patients. These were detected in the gastrointestinal tract (47.3%), bones (15.8%), the respiratory system (10.5%) and the urinary tract (5.3%).

### Diagnostic value

Final IE diagnosis was established in 14 (35%) patients, 2 patients had isolated LDI, and the remaining 24 patients were classified as IE-negative through the complete follow-up observation period. The most common type of endocarditis was CDRIE, which accounted for 64.3% cases, followed by 21.4% of PVE and 14.3% of NVE. Thirteen (32.5%) patients were operated on followed by antimicrobial therapy (12 procedures of complete implantable device removal, 3 valve replacement surgeries, 1 valve repair procedure), 1 patient was treated with antimicrobial therapy alone, and 2 patients with other disease-specific surgical procedures; 24 patients received no treatment.

**Fig. 2:**
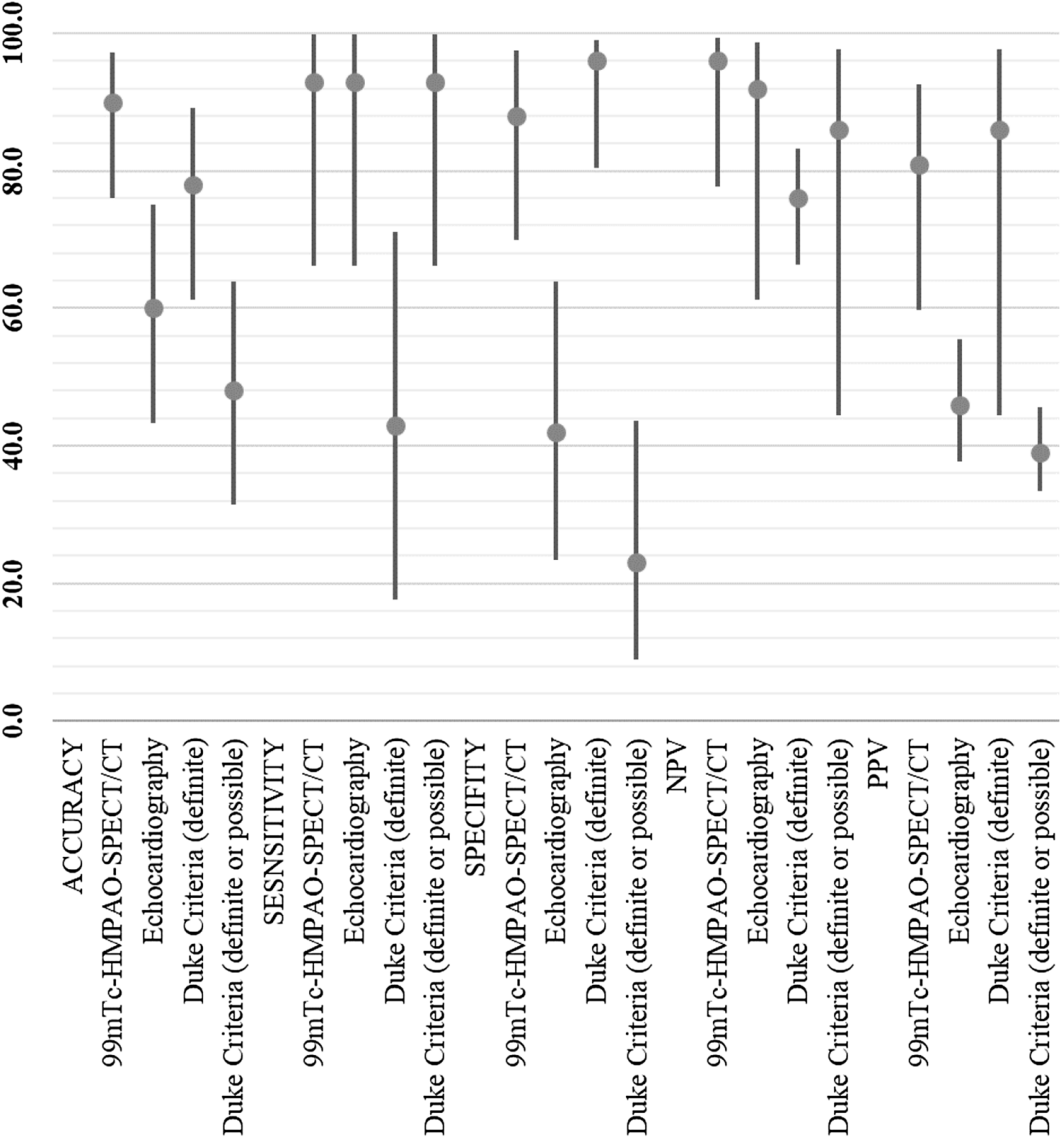
Comparison of TTE, 99mTc-HMPAO-SPECT/CT and modified Duke Criteria diagnostic value in IE suspicion evaluation. Accuracy, sensitivity, specificity, PPV, NPV are shown as values (%) with 95% confidence intervals

The results of endocarditis assessment by TTE, 99mTc-HMPAO-SPECT/CT and modified Duke Criteria are shown in Table 2 and Fig. 2. For IE diagnosis, 99mTc-HMPAO-SPECT/CT was characterized by 90% accuracy, 0.79 Cohen’s kappa coefficient, 93% sensitivity, 88% specificity, 96% NPV and 81% PPV. In this group, TTE was 60% accurate and the kappa value was 0.29. The diagnostic value of TTE in identifying IE was characterized by 93% sensitivity, 42% specificity, 92% NPV and 46% PPV.

**Table 2:** Comparison of TTE, 99mTc-HMPAO-SPECT/CT, and modified Duke Criteria results in IE suspicion evaluation

Type of test	Result for IE	Patients with IE (n = 14)	Patients without IE (n = 26)	Kappa (95% CI)	Accuracy (95% CI)	Sensitivity (95% CI)	Specificity (95% CI)	NPV (95% CI)	PPV (95% CI)
99mTc-HMPAO-SPECT/CT	Positive	13 (93%)	3 (12%)	0.79 (0.59–0.98)	0.90 (0.76–0.97)	0.93 (0.66–0.99)	0.88 (0.7–0.98)	0.96 (0.78–0.99)	0.81 (0.6–0.93)
	Negative	1 (7%)	23 (88%)						
TTE	Positive	13 (93%)	15 (58%)	0.29 (0.07–0.50)	0.60 (0.43–0.75)	0.93 (0.66–0.99)	0.42 (0.23–0.63)	0.92 (0.61–0.99)	0.46 (0.38–0.55)
	Negative	1 (7%)	11 (42%)						
Modified Duke Criteria (definite category)	Positive	6 (43%)	1 (4%)	0.44 (0.16–0.72)	0.76 (0.66–0.82)	0.43 (0.18–0.71)	0.96 (0.8–0.99)	0.76 (0.66–0.83)	0.86 (0.44–0.98)
	Negative	8 (57%)	25 (96%)						
Modified Duke Criteria (definite and possible categories)	Positive	13 (93%)	20 (77%)	0.12 (0–0.29)	0.48 (0.32–0.64)	0.93 (0.66–0.99)	0.23 (0.09–0.44)	0.86 (0.44–0.98)	0.39 (0.34–0.46)
	Negative	1 (7%)	6 (23%)						

Overall for IE, modified Duke Criteria (when a positive result for the test was defined as a definite category) had 76% accuracy and 0.44 kappa. This method had 43% sensitivity, 96% specificity, 76% NPV and 86% PPV. However, when positive results were defined as both definite and possible categories, there was 48% accuracy, 0.12 kappa, 93% sensitivity, 23% specificity, 86% NPV and 39% PPV.

Out of all of the diagnostic methods, radiolabelled leucocyte scintigraphy had the highest accuracy and NPV for IE diagnosis. 99mTc-HMPAO-SPECT/CT was characterized by a statistically significantly higher Cohen’s kappa coefficient, accuracy, specificity and PPV than TTE. The application of scintigraphy in IE diagnostic workup reduces the false-positive results rate in comparison to TTE (3 patients vs. 15 patients).

Compared to modified Duke Criteria, when a positive result for the test was defined as including both definite and possible categories, 99mTc-HMPAO-SPECT/CT had statistically significantly higher Cohen’s kappa coefficient, accuracy, specificity and PPV. When a positive result was defined solely as a definite category, then scintigraphy and modified Duke Criteria diagnostic parameters did not differ significantly; however, 99mTc-HMPAO-SPECT/CT was characterized by a lower rate of false-negative results (1 patient vs. 8 patients).

## Discussion

Due to the on-going change in IE epidemiology, the affected patients are older, have more comorbidities and intracardiac devices [15–17]. Establishing an IE diagnosis is a complex, multi-disciplinary process. Delayed diagnosis and treatment can lead to complications and worse clinical outcomes [18–22]. Due to the fact that 1-year mortality from IE has not improved in over two decades, it is crucial to develop more accurate diagnostic tools [23, 24]. As previously shown, 60% of blood cultures were negative [25, 26]. Nuclear medicine may provide opportunities for personalized evaluation in order to choose the best therapeutic strategy [27, 28]. Nonetheless, data on the diagnostic performance of 99mTc-HMPAO-SPECT/CT is very limited [8, 11–14]. Systematic reviews have shown that it has 64–90% sensitivity, 36–100% specificity, 85–100% PPV and 47–81% NPV [29]. However, the most recent state of art meta-analysis on the role of nuclear imaging in IE identified three studies with a total of 207 patients who had radiolabelled leucocytes scintigraphy. The pooled sensitivity and specificity of 99mTc-HMPAO-SPECT/CT was 86% and 97%, respectively [30]. These findings are consistent with our results, which showed 93% sensitivity and 88% specificity.

Landmark IE studies investigating the diagnostic value of either 99mTc-HMPAO-SPECT/CT or positron emission tomography with fluorodeoxyglucose (18F-FDG PET) used clinical follow-ups as their reference standard [30]. Although this methodology has some inherent flaws, it is believed to be accurate by leading specialists in this field in the context of IE characterization. Histopathological examination is considered to be the ‘gold standard’ for IE diagnosis [6]. Still, including histopathology in a study protocol also has its potential pitfalls and difficulties. Therefore, so far, no validation of nuclear imaging has been performed using histopathological examination.

99mTc-HMPAO-SPECT/CT is reported to be particularly helpful in patients with a high clinical suspicion but previously inconclusive echocardiographic findings, and for differential diagnoses between septic and sterile vegetation, when echocardiographic, laboratory, and clinical tests provide contradictory results [12]. Similarly, in this study 99mTc-HMPAO-SPECT/CT was characterized by significantly higher specificity as well as a lower number of false-positives compared to TTE (3 vs. 15). Since 99mTc-HMPAO-SPECT/CT provides data on current radiolabelled leucocyte accumulation in inflammatory lesions, it was especially useful in echocardiographic lesion differentiation for those with on-going infections and inactive ones.

Transthoracic echocardiography has several advantages, such as wide-spread accessibility, low cost, high safety profile, possibility of both anatomical and functional assessment, which makes it the imaging modality of choice in majority of cardiac conditions, including IE. Even tough TEE is recommended (class I B) in selected groups of patients (with negative/non-diagnostic TTE, prosthetic heart valve or an intra-cardiac device) and is characterized with better diagnostic accuracy than TTE (sensitivity for the diagnosis of vegetation in NVE and PVE is for TEE respectively 96% and 92%), it has also several following limitations: semi-invasive character, requirement of patient’s cooperation or even sedation in selected patients or instable patients [6]. Moreover, both normal TTE and TEE do not completely exclude early stages of infection. In contrary, 99mTc-HMPAO-SPECT/CT is available in highly specialized centers, is far more expensive and study protocol is 24-h-long. Another drawback is radiation as a result of both CT and radioisotope administration.

Modified Duke Criteria, although of paramount importance, have significant limitations, such as low sensitivity for PVE diagnosis [31]. Moreover, 30% of patients with subsequently proven IE are labelled as merely ‘possible IE’ [32, 33]. A reduction in the rate of misdiagnosed IE, previously classified as ‘possible IE’, contributes to the added value of 99mTc-HMPAO-SPECT/CT to the IE diagnostic process [6]. In this study, 26 (65%) patients were classified as ‘possible IE’. In this subgroup, 7 patients (27%) were diagnosed with IE, and, in all those cases, 99mTc-HMPAO-SPECT/CT results were true positives.

99mTc-HMPAO-SPECT/CT is characterized by high specificity in perivalvular regions for active infective processes [34]. It has higher specificity for the diagnosis of PVE than 18F-FDG PET, a modality especially useful in IE diagnostic workup [35–40]. However, 99mTc-HMPAO-WBC accumulation may be low in drained or encapsulated abscesses, or in the case of infection with non-pyogenic bacteria [27]. We observed one case of a false negative 99mTc-HMPAO-SPECT/CT result in a perivalvular abscess in the course of PVE. On the other hand, there were three false-positive scintigraphy results; in one patient, this was most probably related to an autoimmune disorder.

The diagnosis of IE based on standard medical tools without nuclear imaging can be accurate, yet it is very difficult to assess the precise localization and range of the infection. It is especially difficult given the fact that most patients with suspected IE in clinical practice have multiple comorbidities, have undergone multiple cardiac invasive procedures, and often have either a prosthetic valve or an implantable device. In this study, 99mTc-HMPAO-SPECT/CT was useful not only in identifying IE, but also in delimiting the scope of the infection, which is crucial for choosing the appropriate therapy for patients. In this group, none of patients who had 99mTc-HMPAO-SPECT/CT results consistent with LDI developed CDRIE.

99mTc-HMPAO-SPECT/CT also allows for the detection of IE complications such as septic embolic events [6, 41]. 30% of patients with IE have clinical signs of embolization [42]. We detected extracardiac inflammatory foci in 47.5% of scans. This rate is consistent with results from the largest study evaluating the diagnostic value of 99mTc-HMPAO-SPECT/CT (131 patients), where septic emboli were detected in 41% of patients [12].

## Limitations

All patients enrolled to the study were Caucasian. This project was performed in a single department of nuclear medicine. Study results need to be validated in a larger, multicentre study.

## Conclusions

In patients with suspected IE, 99mTc-HMPAO-SPECT/CT provides a lower number of false-positive results, and significantly higher accuracy, specificity and PPV than TTE. Furthermore, this technique helps to differentiate IE echocardiographic morphologic lesions—those with on-going infections and inactive ones. In addition, the use of 99mTc-HMPAO-SPECT/CT leads to a reduction by 27% of the rate of misdiagnosed IE classified in the ‘possible IE’ category by modified Duke Criteria. 99mTc-HMPAO-SPECT/CT may be an invaluable tool not only in diagnosing IE, but also in defining its localization and range, which is particularly important in differentiating between CDRIE and LDI. Overall, 99mTc-HMPAO-SPECT/CT seems to be a useful technique within the IE diagnostic pathway; nevertheless, in future, multicentre studies will be needed for further evaluation of this method.
